# Effectiveness of a Community-Based Individualized Lifestyle Intervention Among Older Adults With Diabetes and Hypertension, Tianjin, China, 2008–2009

**DOI:** 10.5888/pcd11.120333

**Published:** 2014-05-15

**Authors:** Ruijun Yu, Lijing L. Yan, Hanliang Wang, Liang Ke, Zhou Yang, Enying Gong, Hui Guo, Jun Liu, Yuting Gu, Yangfeng Wu

**Affiliations:** Author Affiliations: Ruijun Yu, Jun Liu, Tianjin Health Insurance Research Association, Tianjin, China; Lijing L. Yan, The George Institute for Global Health at Peking University Health Science Center, Beijing, China, and Northwestern University, Chicago, Illinois; Hanliang Wang, Zhou Yang, Health Promotion Working Committee, National Center for Medical Education Development, Beijing, China; Liang Ke, Enying Gong, Hui Guo, The George Institute for Global Health at Peking University Health Science Center, Beijing, China; Yuting Gu, Department of Rehabilitation, The First Affiliated Hospital of Tianjin Chinese Traditional Medicine University, Tianjin, China.

## Abstract

**Introduction:**

Though diet and exercise modification is effective in preventing diabetes and hypertension, community-based models for lifestyle intervention for managing these conditions that are practical and effective are few.

**Methods:**

A community-based lifestyle intervention trial was conducted in 5 community clinics in Tianjin, China. Trained physicians used energy monitors and software as tools to provide eight individualized lifestyle consultation sessions (zhiji management) to 273 residents with mild hypertension (including prehypertension) or diabetes (including prediabetes). The recruitment was based on a waitlist control design. The early group (n = 175) received the 3-month intervention and the late group served as controls; afterward, the early group was followed up while the late group received the 3-month intervention. Selected characteristics between the 2 groups were compared by χ^2^ tests, continuous variables paired *t* tests, and independent *t* tests.

**Results:**

Compared with baseline, the intervention significantly increased effective (3–6 metabolic equivalents and >6 minutes) physical activity by 54.6 kilocalories per day (*P* < .01) and decreased total dietary intake by 328.5 kilocalories per day (*P* < .01). The net differences between early group (intervention) and late group (control phase) were significant (*P* < .01) for weight, waist circumference, systolic and diastolic blood pressure, 2-hour postprandial glucose, and hemoglobin A1c.

**Conclusion:**

This community-based lifestyle zhiji management program produced short-term beneficial changes in activity, diet, and clinical parameters in patients with mild diabetes or hypertension. Larger and longer trials are needed to fully evaluate the effectiveness and feasibility of this model.

## Introduction

Diabetes and hypertension are major global public health problems with high and increasing prevalence. They both have serious cardiovascular, renal, and other health consequences and share common lifestyle risk factors. Scientific evidence shows that lifestyle modifications such as having a healthful dietary pattern and being physically active could prevent hypertension or diabetes and reduce the risk of developing these conditions among high-risk individuals not yet having these diseases (1,2). Some translational studies show how these lifestyle modifications can be adopted in a community setting (3–5). However, few studies have examined whether these lifestyle modifications (with or without other medical therapies) can be effective for patients with hyperglycemia and hypertension (6–8).

According to the noncommunicable diseases surveillance report, the prevalence of hypertension and diabetes was 33.5% and 9.7%, respectively, in 2010 among Chinese adults (9). Chinese health care reform mandated the prevention and management of hypertension and diabetes as national public health priorities and delegated such responsibilities mainly to primary care providers and community health care centers (10). Therefore, evidence from community-based studies on lifestyle modification programs for hypertension and diabetes prevention and control are urgently needed.

To address the increasing burden of diabetes and hypertension and the lack of evidence on lifestyle intervention programs for the management of diabetes and hypertension among patients, we conducted a community-based individualized lifestyle intervention study in China. The intervention was based on well-established theories including energy balance in terms of physical activity and dietary energy intake. The aim of our study was to investigate and evaluate the effectiveness of this lifestyle intervention program delivered by community physicians on managing hypertension and diabetes and improving health indicators in a community health care setting in China. The hypothesis for this study is that the 3-month intensive intervention will significantly improve the following biophysical indicators (weight, waist circumference, systolic blood pressure [SBP], diastolic blood pressure [DBP], fasting glucose level, and 2-hour postprandial glucose) in comparison with the control group.

## Methods

**Figure 1:**
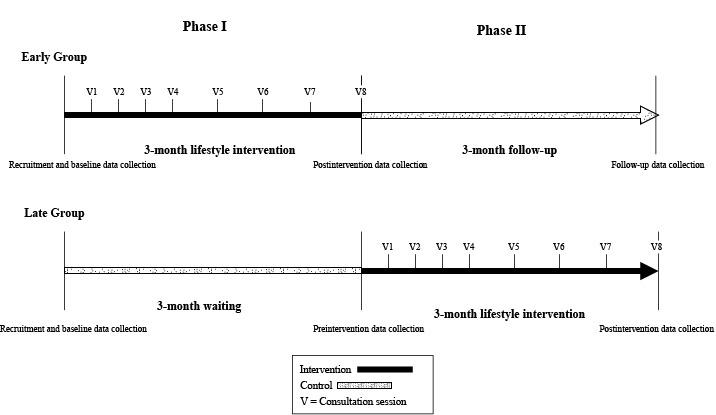
Study design for individualized lifestyle intervention in primary care facilities, Tianjin, China, 2008–2009.

A community-based controlled trial was conducted from September 2008 to March 2009 in 5 local community health clinics in Tianjin City, China. The recruitment was based on a waitlist control design and participants were assigned to early group or late group. In each clinic, the early group volunteers who were eligible and interested in participating in the intervention program were assigned to the early group and other eligible participants were assigned to the late group. During phase I of the study, the early group received a 3-month intensive lifestyle intervention program on improving physical activity and dietary patterns while the late group received usual standard health care, serving as controls; during phase II of the study, the early group received a 3-month follow-up without any intervention while the late group received the 3-month intensive lifestyle intervention (Figure 1).

The study participants were recruited consecutively by community physicians in the local clinics during usual visits. All patients were aged 45 to 75 years. The inclusion criteria were fasting blood glucose between 5.6 and 14 mmol/L, postprandial blood glucose between 7.8 and 17 mmol/L, SBP from 120 to 180 mm Hg, or DBP from 80 to 105 mm Hg. The exclusion criteria were inability to perform physical activity independently, mental illness, or other severe medical conditions such as diabetes nephropathy and hypertensive encephalopathy. Initially, there were 287 study participants registered and assigned to the 2 study groups. Fourteen patients in the late group withdrew 2 weeks before baseline data collection, leaving 273 study participants (early group n = 175; late group n = 98). No participant withdrew during the intervention.

### Intervention

The intervention is called “zhiji management”; zhiji is a Chinese word meaning “bosom friends.” Zhiji management is a 3-month lifestyle intervention program designed to quantify and balance dietary energy intake and energy consumption from physical activity and to provide practical suggestions on positive and safe lifestyle changes. All participants kept their medical treatment as usual.

Each participant was supplied with an electronic accelerometer-like energy consumption monitor (Zhiji Energy Monitor, YHKY Ltd, Beijing, China) to wear all day long except when sleeping or showering. The monitor recorded physical activity in terms of duration in minutes and intensity measured by metabolic equivalent (MET) of the activities (11). Effective physical activity was defined as any activity between 3 and 6 METs and lasting at least 6 minutes. Because most study participants were chronically ill older people, we adopted a shorter duration than the 10 minutes recommended in the US guideline (12). Participants were also instructed to keep a dietary diary at least 2 days a week, a workday (Monday through Friday) and a weekend day.

Health-related individualized consultations were provided during the 3-month intervention. Trained community physicians in each clinic, aided with customized computer software designed for the zhiji intervention, met study participants individually for weekly consultation sessions in the first month and biweekly sessions in the following 2 months, for a total of 8 sessions (Figure 1). During each 35- to 40-minute session, the community physicians downloaded physical activity data from the accelerometer to the computer in the clinic and converted the information from the dietary diary to computerized data. The software produced a detailed physical activity and dietary intake status, illustrated by charts and tables and individualized “prescriptions” for lifestyle modification. Community physicians explained these results to each participant and encouraged the participant to engage in appropriate, safe, and effective physical activity and to follow dietary recommendations in line with the Chinese dietary guidelines (13). The physicians also suggested ways to balance energy intake and consumption. The computer printouts with measurable targets and the prescription were given to the participants to take home as visual aids and reminders.

### Measurements

At the time of recruitment, marital status, educational level, employment status, and self and family medical history were collected through a standardized questionnaire administered by community physicians, and body weight and height were measured. During each of the 8 interventional sessions, body weight, waist circumference, SBP and DBP were measured by community physicians. Body weight was measured by an electronic scale (Xihen, Wuxi Scale Co, Wuxi City, Jiangsu Province, China) with subjects wearing light indoor clothing and no shoes. Waist circumference was measured at a level midway between the lowest rib and the iliac crest by a measuring tape. After a 5-minute rest, blood pressure was measured twice with an electronic blood pressure meter (Omron HEM-7200, Omron (China) Co, Ltd, Dalian City, Liaoning Province, China) and the average values were used in all analyses. In addition to these measurements, the early group had 2 additional measurements in the second and third month during the 3-month postintervention follow-up period and the late group had 2 measurements in the first and second month. For participants with diabetes only, blood tests of fasting glucose, 2-hour postprandial glucose, and glycated hemoglobin (HbA1c) were performed during the intervention and follow-up on a voluntary basis.

### Statistical analysis

We compared selected characteristics of the early and late groups (percentages for categorical variables; mean and standard deviation for continuous variables). Within-group (preintervention and postintervention) and between-group differences in categorical variables were tested by χ^2^ tests and continuous variables with paired *t* tests and independent *t* tests. Intention-to-treat analysis using the method of “last observation carried forward” was applied for all measures. Multivariable analysis was conducted to test the effect of demographic characteristics on the outcomes. All data analyses were performed by using SPSS version 15 for Windows (SPSS Inc, Chicago, Illinois).

### Ethical consent

The study followed the principles of the Declaration of Helsinki. This study was approved by the Project Examination Committee of the Tianjin Department of Labor and Social Security. All participants gave their written informed consent before participating in the study.

## Results

The average age of the 273 participants was 60.6 years; 56% were female, 92% were married, 32% had a college education, and 64% were retired; and 54% had known diabetes and 68% had known hypertension (Table 1). There was no significant difference between the 2 groups in any of these characteristics (all *P* values > .05).

**Table 1:** Comparison of Baseline Characteristics Among Participants in an Individualized Lifestyle Intervention, Tianjin, China, 2008–2009^a^

Characteristic	Total (N = 273)	Early Group (n = 175)	Late Group (n = 98)
**Age, y, mean (standard deviation**)	60.6 (10.1)	60.9 (10.2)	60.1 (10.0)
**Sex**			
Male	119 (44)	77 (44)	42 (43)
Female	154 (56)	98 (56)	56 (57)
**Marital status^b^**			
Married	169 (92)	130 (92)	39 (95)
Other	14 (8)	12 (8)	2 (4)
**Education^b^**			
Primary school or lower	8 (5)	7 (5)	1 (2)
Middle or high school	115 (63)	87 (62)	28 (68)
College or university	59 (32)	47 (33)	12 (29)
**Occupational status^b^**			
Employed	61 (34)	49 (35)	12 (31)
Retired	116 (64)	90 (63)	26 (67)
Other	4 (2)	3 (2)	1 (3)
**Diabetes**	147 (54)	94 (54)	53 (54)
**Hypertension**	185 (68)	124 (71)	61 (62)

a All variables are reported as n (%), unless otherwise noted. All *P* values for comparison between the early and late groups were > .05.

b Number of missing values: marital status, 90; education, 91; and occupational status, 92.

### Changes in physical activity and dietary intake

After the intervention, daily effective physical activity increased significantly (*P* < .001) in total energy expenditure (54.6 kcal) and duration of exercise (9.6 min) (Table 2). Daily total energy intake decreased significantly (*P* < .001) (328.5 kcal) with a small increase in total energy from protein and a small decrease from fat. Results were generally consistent between the early and the late group for the 3 months of the intervention phase.

**Table 2:** Physical Activity and Dietary Intake Before and After a 3-Month Lifestyle Intervention, Tianjin, China, 2008–2009^a^

Characteristic	Early Group (n = 175)				Late Group (n = 98)				Total (N = 273)			
	Pre- intervention	Post- intervention	Change	*P* Value^b^	Pre- intervention	Post- intervention	Change	*P* Value^b^	Pre- intervention	Post- intervention	Change	*P* Value^b^
**Physical activity**												
Total physical activity, kcal	408.8 (188.2)	394.6 (194.7)	−14.2	.34	291.8 (148.5)	366.9 (193.9)	75.1	<.001	366.8 (183.5)	384.6 (194.5)	17.8	.13
Effective physical activity, kcal	134.8 (112.1)	183.6 (129.3)	48.8	<.001	113.9 (97.9)	178.7 (129.3)	64.8	<.001	127.3 (107.5)	181.9 (129.1)	54.6	<.001
Effective physical activity times, min	26.7 (22.3)	35.0 (24.3)	8.3	<.001	22.1 (18.5)	34.1 (25.2)	12.0	<.001	25.1 (21.1)	34.7 (24.6)	9.6	<.001
**Dietary intake**												
Total energy from diet, kcal	2,224.4 (592.2)	1,933.4 (328.3)	−291.0	<.001	2,183.8 (423.6)	1,788.6 (234.7)	−395.2	<.001	2,209.9 (537.3)	1,881.4 (305.7)	−328.5	<.001
Total protein, % energy	15.8 (3.4)	17.1 (3.4)	1.3	<.001	16.8 (3.0)	15.9 (2.4)	−0.9	.005	16.1 (3.3)	16.7 (3.1)	0.6	.009
Total fat, % energy	27.1 (7.3)	26.2 (5.9)	−0.9	.13	27.6 (5.2)	26.3 (4.0)	−1.3	.005	27.3 (6.6)	26.2 (5.3)	−1.1	.010
Total carbohydrate, % energy	55.4 (10.4)	55.5 (8.9)	0.1	.92	55.6 (6.9)	57.8 (5.0)	2.2	.001	55.5 (9.3)	56.3 (7.8)	0.8	.097

a Values shown are mean (standard deviation) unless otherwise noted.

b Paired *t* test.

### Net differences in biophysical markers between intervention and control groups

During phase I, body weight, waist circumference, SBP, and DBP decreased significantly (*P* < .001) in the early group that received the 3-month intervention, and most indicators worsened in the late group that served as controls during the same phase (Table 3). The net between-group reductions were substantial and significant for weight (−2.6 kg), waist circumference (−2.9 cm), SBP (−10.9 mm Hg), and DBP (−4.0 mm Hg); all *P* values < .001. Results were similar for the subgroup of patients with diabetes for whom we had glucose and HbA1c data.

**Table 3:** Comparisons of Biophysical Outcomes Between the Intervention and Control Groups Among Participants in an Individualized Lifestyle Intervention, Tianjin, China, 2008–2009^a^

Characteristic	Early (Intervention ) Group				Late (Control) Group				Difference in Change^c^	*P* Value^b^
	Preintervention	Postintervention	Change	*P* Value^b^	Baseline	End	Change	*P* Value^b^		
**Total sample (n = 175 for early group; n = 98 for late group)**										
Weight, kg	69.9 (10.8)	68.7 (10.7)	−1.2	<.001	70.6 (11.4)	72.0 (11.9)	1.4	<.001	−2.6	<.001
Waist circumference, cm	92.7 (10.1)	90.5 (9.8)	−2.2	<.001	91.0 (11.4)	91.7 (11.2)	0.7	<.001	−2.9	<.001
Systolic blood pressure, mm Hg	132.1 (15.8)	127.7 (12.7)	−4.4	<.001	130.0 (16.5)	136.5 (17.1)	6.5	<.001	−10.9	<.001
Diastolic blood pressure, mm Hg	80.0 (10.0)	77.9 (8.3)	−2.1	<.001	80.1 (8.9)	82.0 (7.4)	1.9	.010	−4.0	<.001
**Subsample of patients with diabetes (n = 94 for early group; n = 53 for late group)**										
Fasting glucose, mmol/L	7.3 (2.4)	7.0 (2.0)	−0.3	.16	6.9 (1.8)	7.5 (1.7)	0.6	.01	−0.9	.08
2-hour postprandial glucose, mmol/L	10.6 (3.8)	8.6 (1.9)	−2.0	<.001	9.5 (3.1)	9.8 (3.0)	0.3	.54	−2.3	<.001
HbA1c, %^d^	6.8 (1.2)	6.5 (1.0)	−0.3	.009	6.7 (1.1)	6.9 (1.0)	0.2	.097	−0.5	.008

Abbreviation: HbA1c, glycated hemoglobin.

a Values shown are mean (standard deviation) unless otherwise noted.

b Paired *t* test.

c Difference between change in intervention group and change in the control group.

d Intention to treat analysis using the method of “last observation carried forward” (n = 4 in diabetes in the early group, n = 2 in diabetes in the late group).

Multivariable analysis was conducted to control for other potential dependence-associated factors. The outcome variables were the 7 biophysical indicators, and the independent variables included group (intervention vs control), the outcome variable’s corresponding baseline level, sex, marital status, education, and occupational status. The multivariable results confirmed that the net differences between the early group (intervention phase) and late group (control phase) were highly significant for all the following biophysical indicators assessed: weight, waist circumference, SBP, DBP (all *P* values < .001). Among patients with diabetes, the intervention significantly improved 2-hour postprandial glucose and HbA1C results (*P* < .01), but the effect on fasting glucose levels was borderline significant (*P* = .08).

### Session-by-session results and follow-up data

**Figure 2:**
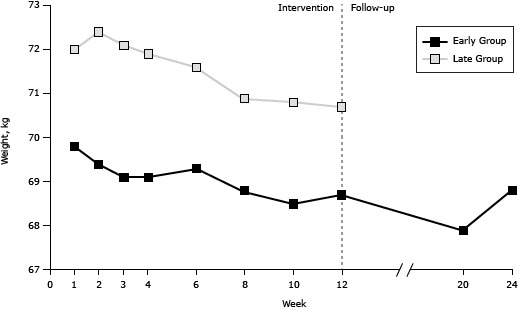

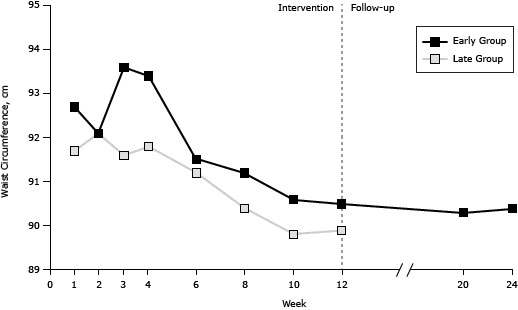

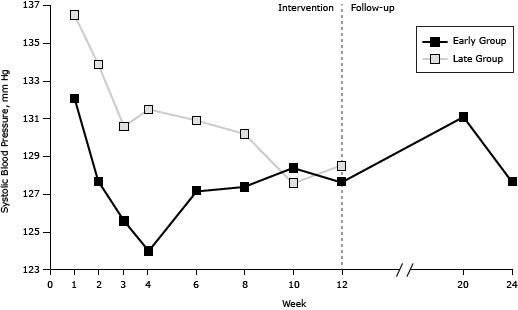

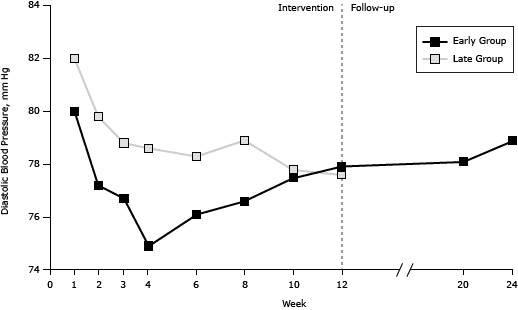
Session-by-session results for weight, waist circumference, systolic blood pressure, and diastolic blood pressure among participants in an individualized lifestyle intervention, for both the early and the late group interventions and during follow-up for the early group Tianjin, China, 2008–2009. Total sample: early group n = 175, late group n = 98. **Table:** 

During the intervention, a generally consistent pattern of gradual improvement was observed for all markers and both intervention groups (Figure 2). No further improvement was observed during the follow-up in the early group for whom such data were available. Nevertheless, the effect of the intervention was maintained for all markers assessed. Additional analyses based on participants without any missing values for all sessions (early group = 135, late group = 78) revealed consistent results.

Week	Weight, kg		Waist Circumference, cm		Systolic Blood Pressure, mm Hg		Diastolic Blood Pressure, mm Hg	
	Early Group	Late Group	Early Group	Late Group	Early Group	Late Group	Early Group	Late Group
**Intervention**								
1	69.8	72.0	92.7	91.7	132.1	136.5	80.0	82.0
2	69.4	72.4	92.1	92.1	127.7	133.9	77.2	79.8
3	69.1	72.1	93.6	91.6	125.6	130.6	76.7	78.8
4	69.1	71.9	93.4	91.8	124.0	131.5	74.9	78.6
6	69.3	71.6	91.5	91.2	127.2	130.9	76.1	78.3
8	68.8	70.9	91.2	90.4	127.4	130.2	76.6	78.9
10	68.5	70.8	90.6	89.8	128.4	127.6	77.5	77.8
12	68.7	70.7	90.5	89.9	127.7	128.5	77.9	77.6
**Follow-up**								
20	67.9	NA	90.3	NA	131.1	NA	78.1	NA
24	68.8	NA	90.4	NA	127.7	NA	78.9	NA

Abbreviation: NA, not applicable.

## Discussion

This study was one of the first community-based lifestyle modification studies in China among people with mild diabetes (including prediabetes) or hypertension (including prehypertension) instead of among people who did not have the conditions but were at high risk of developing them. Significant increases in effective physical activity and decreases in total dietary energy intake were achieved after the 3-month zhiji intervention led by community physicians that included computer-aided individualized consultation on lifestyle during 8 sessions. The intervention group had significantly greater net reductions in all biophysical indicators than did the control group. The improvements in these markers were maintained during the approximately 3-month follow-up period.

### Effect on lifestyle modification

The intensive zhiji intervention led by community physicians was effective in encouraging the participants to adopt and maintain a healthier lifestyle in terms of physical activity and diet. This finding is consistent with the findings of most other studies of this kind, including the Diabetes Prevention Program (DPP), the study by Lindström et al, and other studies (2,8,14–17). Also, the Look AHEAD (Action for Health in Diabetes) research group found that lifestyle intervention was effective among diabetes patients (6). In addition to these findings, our study added further evidence showing that a community-based intensive lifestyle intervention is effective at getting patients with mild diabetes (including prediabetes) or hypertension (including prehypertension) to adopt and maintain a healthier lifestyle.

We found that total physical activity did not increase but total effective physical activity significantly increased through the intervention. In this intervention program, the concept of effective physical activity was emphasized, with patients instructed to engage in physical activities that reach a certain level of intensity (at least 3 METs) to achieve health benefits but are not too strenuous to be unsafe (more than 6 METs). The instantaneous quantification of activity intensity in MET was made possible by the energy consumption monitor, which had more sophisticated functions than pedometers.

The zhiji intervention program in this study shares some common features with other programs such as the DPP (2), including being individualized, being delivered by a case manager (in our study, by community physicians), and having frequent contact between physician and patient. However, unlike the DPP or other studies (2,4,18–20) that used goal-based behavioral intervention and a structured diet and physical activity plan for the core curriculum, our study adopted a “free choice” lifestyle modification model, operating under well-established guidelines. This model may have empowered patients’ self-management skills through enabling them to select the most comfortable and easy to adopt behavioral changes.

### Effect on biophysical markers

Significant reductions were observed in anthropometrics (weight and waist circumference), SBP, DBP, and glucose measures. These findings were consistent with those of previous studies in which intensive individual-based dietary and physical activity modification programs produced similar mean losses in weight (17,19,21,22), waist circumference (4,17,19,23), and blood pressure (4,16,24,25). Most previous studies were conducted among individuals at high risk of developing diabetes or hypertension. Our study demonstrated that intensive lifestyle modification in the community setting could also lead to improvements in these markers in patients with mild diabetes (including prediabetes) and hypertension (including prehypertension).

### Chronic care model

The chronic care model provides a framework for a systematic approach with 6 key elements that are critical to effective management of chronic diseases (26–29). The 6 elements are the community, the health system, self-management support, delivery system design, decision support, and clinical information systems. Although not explicitly designed on the basis of this model, this intervention program incorporated all 6 of those elements.

### Limitations and strengths

Our study has several limitations. First, this trial was not randomized. Nevertheless, the late group served as controls during phase I of the study, and provided a way to assess net changes in many measures instead of only pre–post comparison. In addition, both groups had similar demographic characteristics. Second, follow-up data were limited to the early group and lasted only 3 months. Therefore, the long-term effect of the 3-month intervention could not be assessed. Third, our study sample was derived from volunteer participants in 5 community clinics, and it was possible that these participants were not representative of all patients with hypertension or diabetes. Nevertheless, no systematic differences were detected between the early and the late group, making them generally comparable. Lastly, there may be contamination for the late group while serving as controls because they lived in the same community with the early group participants undergoing intervention. However, the outcomes improved in the early group while most of them worsened in the late group, suggesting that contamination, if any, was limited.

Our study also has several strengths. First, the intervention program was carefully designed to use, and modify when appropriate, established physical activity and dietary guidelines. Second, the program was well accepted by study participants, and completeness of data collection for behavioral and clinical measures was better than they were for most other studies. Third, our study provides important evidence regarding the effectiveness of these interventions at not only preventing diabetes and hypertension but also at managing these conditions. After the study was complete, the zhiji program was implemented in more communities in several cities in China such as Beijing, other areas of Tianjin, and Xi’an.

### Conclusion

The burden of diabetes and hypertension has been rising fast in developing countries. Previous evidence on the effect of lifestyle modification focused on prevention of hypertension and diabetes among people who did not have these conditions but were at high risk of developing them. Highlights of the innovative features of our intervention included training and delivery of the intervention by community physicians, supplying participants with energy monitors, emphasizing effective physical activity, using computer software for electronic decision support and for printing a tailored intervention program for each participant, and empowering patients with self-management skills and community support. This community-based individualized lifestyle intervention program produced short-term beneficial changes in activity, diet, and clinical parameters in patients with diabetes or hypertension. Its cost-effectiveness or feasibility in scaling up into more communities is not yet clear. Future large-scale research with longer follow-up is needed to fully evaluate the cost-effectiveness and feasibility of such a lifestyle intervention among patients with diabetes, hypertension, or other chronic disease.
